# Complete mitochondrial genome and phylogenetic implications of the rainbow bitterling *Acheilognathus tonkinensis* (Cypriniformes: Cyprinidae)

**DOI:** 10.1080/23802359.2018.1524278

**Published:** 2018-10-26

**Authors:** Man Zhang, Kaicheng Wang, Liangkui Zhang, Zongjian Tang, Shengping Zhong, Hongyu Tan, Lin Wu, Xiuli Chen, Guangping Cheng

**Affiliations:** a Guangxi Colleges and Universities Key Laboratory of Aquatic Healthy Breeding and Nutrition Regulation, College of Animal Science and Technology, Guangxi University, Nanning, China;; b Key Laboratory of Marine Biotechnology, Guangxi Institute of Oceanology, Beihai, China;; c Guangxi Academy of Fishery Sciences, Nanning, China

**Keywords:** *Acheilognathus tonkinensis*, mitochondrial genome, phylogeny

## Abstract

*Acheilognathus tonkinensis* is a magnificent bitterling fish of genus *Acheilognathus* belonging to the sub-family Acheilognathinae of the family Cyprinidae. In this study, we first determined and described the 16,767 bp mitochondrial DNA sequence of *A. tonkinensis*. The mitogenome encoded 13 protein-coding genes (PCGs), 22 tRNA genes, 12S, and 16S rRNA genes, and a D-loop region. The overall nucleotide composition was 29.4% A, 27.1% T, 17.0% G, and 26.5% C, with a slight AT bias (56.5%). Phylogenetic analysis suggested that *A. tonkinensis* had the closest evolutionary relationship with *A. macropterus*. The availability of mitogenome sequence of *A. tonkinensis* would facilitate species identification of the Acheilognathinae, as well as genetic evaluations for resource conservation and management of this species.

The rainbow bitterling, *Acheilognathus tonkinensis* (Cypriniformes: Cyprinidae), has a wide range in south-east Asia and is mainly distributed in freshwater rivers of central and southern China (Chen et al. 1998). Its unique spawning behavior and brilliant color has made it widely popular and which brought extremely high ornamental and economical value to it. Thus it is likely to be impacted by overfishing, water pollution, and damming. Nowadays, research on this species is largely focused on karyotype, reproductive strategy, and heavy metal accumulation (Arai et al. 1992; Liu et al. 2006; Wang et al. 2017), but little information is available on the mitochondrial sequences. The present study reported the complete mitogenome of *A. tonkinensis* and validated its taxonomic status, which would be conducive to further molecular genetic studies and conservation of this species.

The specimen of *A. tonkinensis* was collected in July 2016 from Wuxuan County, Guangxi, China (23°35′35.29″N, 109°39′45.27″E) and was deposited in Guangxi Colleges and Universities Key Laboratory of Aquatic Healthy Breeding and Nutrition Regulation, Guangxi University. Genomic DNA was isolated from the dorsal muscle by Animal Tissues Genomic DNA Extraction Kit (Solarbio, Beijing, China). The sequence was amplified through conventional and long PCR with fourteen primer pairs, and then sequenced in Sangon Biotech (Shanghai, China). Mitochondrial genome was assembled and annotated by Geneious 10.1.2 (Biomatters Ltd., Auckland, New Zealand) using the *A. macropterus* reference mitogenome (GenBank Accession No. KJ499466.1) (Zhu et al. 2016).

The mitochondrial genome of *A. tonkinensis* was 16,767 bp in length (GenBank Accession No. MH261370) and was A + T-biased (56.5%) with a base composition of 29.4% A, 27.1% T, 17.0% G, and 26.5% C, respectively. It retained the canonical set of 37 genes observed in bony fish mitogenomes, presenting 13 PCGs, 22 tRNA genes, 2 rRNA genes, a putative origin of light-strand replication, and an AT-rich control region (D-Loop). Besides, 12 PCGs were initiated by ATG except *COI* gene used GTG as a start codon. The stop codon TAA was identified in 6 PCGs (*ND1, COI, ATP8, ND4L, ND5* and *ND6*), while the aberrant single-nucleotide termination codon (T) was found in the rest of the coding genes. Two rRNAs (958 bp 12S and 1678 bp 16S) were composed of 54.0% AT, and the D-loop was 1119 bp long with the AT content of 66.6%.

To confirm the phylogenetic place of *A. tonkinensis* in the Acheilognathinae, a maximum likelihood dendrogram was constructed by MEGA 7.0 software based on all PCGs of 17 Acheilognathinae fishes (Kumar et al. 2016). Two species of Barbinae were employed as an outgroup taxa. Evolutionary model was selected by the program PhyML 3.0 (Guindon et al. 2010), where the GTR + G + I substitution model was recognized as the best fit to the data. The distance dendrogram revealed the closest evolutionary relationship of *A. tonkinensis* to the chinese bitterling *A. macropterus* (Figure 1), and the arrangement of taxa within the Acheilognathinae was in accordance with the previously reported results (Chang et al. 2014).

**Figure 1.**
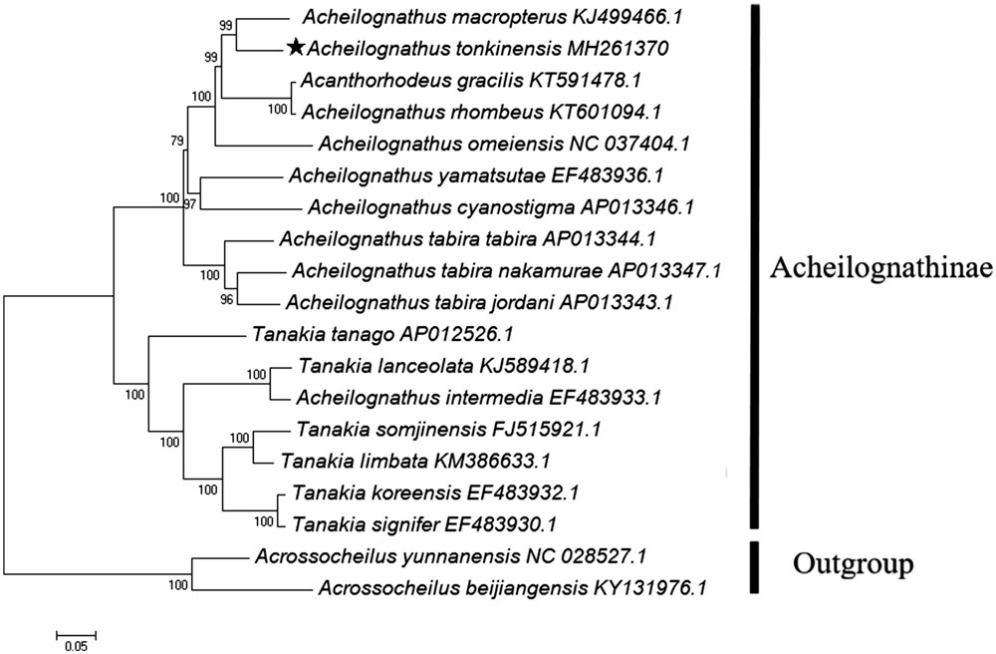
Maximum-likelihood tree (GTR + G + I model) of *Acheilognathus tonkinensis* and 16 related Acheilognathinae species based on 13 concatenated PCGs. Bootstrap values (1000 replicates) are indicated at each node. Two species of Barbinae were utilized as outgroup taxa. The mitogenomic information of *A. tonkinensis* is marked with pentagram.
